# A potential link between gambling addiction severity and central dopamine levels: Evidence from spontaneous eye blink rates

**DOI:** 10.1038/s41598-018-31531-1

**Published:** 2018-09-06

**Authors:** David Mathar, Antonius Wiehler, Karima Chakroun, Dominique Goltz, Jan Peters

**Affiliations:** 1 Department of Psychology, Biological Psychology, University of Cologne, Cologne, Germany; 2 Department of Systems Neuroscience, University Medical Center Hamburg-Eppendorf, Hamburg, Germany; 3 Motivation Brain Behavior, ICM - Hôpital Pitié-Salpêtrière, Paris, France

## Abstract

Accumulating evidence points at similarities between substance use disorders (SUD) and gambling disorder on the behavioral and neural level. In SUD, attenuation of striatal D2/3-receptor availability is a consistent finding, at least for stimulating substances. For gambling disorder, no clear association with striatal D2/3-receptor availability has been unveiled so far. With its presumably negligible dopaminergic toxicity, possible differences in receptor availability in gambling disorder might constitute a vulnerability marker. Spontaneous eye blink rate (sEBR) is discussed as a potential proxy measure for striatal dopamine D2/3-receptor availability. Here we examined sEBR in 21 male problem gamblers and 20 healthy control participants. In addition, participants completed a screening questionnaire for overall psychopathology and self-reported measures of alcohol and nicotine consumption. We found no significant difference in sEBR between gamblers and controls. However, in gamblers, sEBR was negatively associated with gambling severity and positively associated with psychopathology. A final exploratory analysis revealed that healthy controls with low sEBR displayed higher alcohol and nicotine consumption than healthy participants with high sEBR. Although the exact association between dopamine transmission and sEBR is still debated, our findings reveal that sEBR is sensitive to inter-individual differences in gambling disorder severity in problem gamblers.

## Introduction

Placing something valuable at risk with the hope of gaining something of greater value is a popular recreational activity among adults, and is referred to as gambling^1^. Approximately five percent of the population encounter sublinical problematic gambling and around one percent display pathological gambling^2,3^. Gambling disorder has been classified as an addiction disorder in the Diagnostic and Statistical Manual of Mental Disorders (5th ed.; DSM-5; American Psychiatric Association, 2013). This classification is based on accumulating evidence revealing similarities between pathological gambling and substance use disorders (SUDs) in both behavioral observations and underlying neural mechanisms^4–6^. Individuals meeting at least one of the DSM V criteria are commonly referred to as problem gamblers, whereas individuals meeting four or more are referred to as pathological gamblers.

A consistent finding in individuals suffering from addiction to stimulating drugs or alcohol is a dysregulation of dopaminergic transmission within striatal target sites^7^. Patients suffering from SUDs show reduced dopamine D2/3-receptor availability in the striatum^8–12^, constituting a candidate risk factor for the development of SUDs^13^. In monkeys and rodents, attenuated striatal dopamine D2/3-receptor availability prior to drug exposure is related to a more rapid acquisition of drug self-administration^14,15^. Reduced D2/3-receptor modulation of corticostriatal pathways may translate into a higher risk of escalating drug abuse via increased impulsivity^15–17^. D2/3-receptor function is also tightly coupled to learning from negative outcomes^18,19^, a process recently shown to be impaired in cocaine addicts compared with healthy controls^20^.

In gambling disorder, initial evidence for alterations in dopaminergic transmission came from studies that reported increased dihydroxtyphenylacetic (DOPAC) levels in cerebrospinal fluid of gambling addicts, and increased dopamine levels in blood samples of gamblers compared with controls during gambling^21,22^. In addition, dopaminergic medication in Parkinson’s disease patients can induce problem gambling as a side-effect^23^. More recent positron-emission-tomography (PET) studies tested direct associations between gambling disorder and central dopamine transmission. Van Holst *et al*.^24^ found increased striatal dopamine synthesis capacity, as assessed with [^18^F]fluoro-levo-dihydroxyphenylalanine ([^18^F]DOPA) PET, in gamblers compared with controls. Gamblers also showed higher amphetamine induced striatal dopamine release compared with controls^25^. Striatal D2/3-receptor availability was shown to be associated with mood related impulsivity and gambling severity in pathological gamblers, but no differences in striatal D2/3-receptor availability between gamblers and controls in general were observed so far^26–28^.

Spontaneous eye blink rate (sEBR) is discussed as a potential non-invasive proxy measure of striatal dopamine transmission^29^. Initial evidence linking sEBRs to dopamine transmission came from observations in several neurological and psychiatric disorders that relate to alterations in central dopamine regulation such as Parkinson’s disease, schizophrenia and psychosis^30–33^. In line with reduced D2/3-receptor availability, cocaine users^34^ and chronic cannabis consumers^35^ display reduced sEBRs compared to healthy controls. Several pharmacological studies observed a reduction in sEBR following dopamine antagonist administration, and an increase after dopamine agonist administration^36–39^. Complementing these findings, a recent PET study in monkeys found a strong positive correlation between sEBR and D2/3-receptor availability in ventral and parts of dorsal striatum^40^. Consistent with the idea that sEBR measures trait-like differences in dopaminergic transmission, it has good reliability (Cronbach’s Alpha: 0.79–0.85, see Kruis *et al*.^41^). However, two recent studies report opposing findings. Dang *et al*.^42^ found no significant correlation between sEBR and D2/3-receptor availability in midbrain and striatum in humans. In addition, they observed no significant impact of bromocriptine, a dopamine agonist, on participants’ sEBRs. Noteworthy, their subject sample was quite heterogeneous regarding age and body weight, and between 3 and 32 months separated subjects’ sEBR assessment from PET imaging. Quite recently, Sescousse *et al*.^43^ report a negative correlation between sEBR and striatal dopamine synthesis capacity in a mixed sample of healthy controls and gamblers. Thus, it is still debated to what extent, and with which specific aspect of dopaminergic transmission sEBR is associated with. A recent review suggested that current evidence is most supportive of a link between D2/3-receptor availability and sEBR^44^.

A key advantage of sEBR over other methods is that it is affordable and easily obtainable. In the light of the putative link of sEBR and D2/3-receptor availability, it is of considerable clinical interest to explore alterations of sEBR in behavioral addictions such as gambling disorder. Here, we utilize sEBR to examine potentially D2/3-receptor availability modulated group differences between problem gamblers and healthy control participants. According to the PET studies that showed a negative correlation between D2/3-receptor availability in gamblers and impulsivity, and gambling severity, respectively^26–28^, we hypothesize to observe a negative association between gambling severity and sEBR. As psychopathology is known to be related to aberrant central dopamine transmission^45–47^ and recently was shown to correlate with sEBR^33^, we utilize the SCL-90-R questionnaire^48^ as a screening test, and to control for overall psychopathology.

## Results

**Figure 1:**
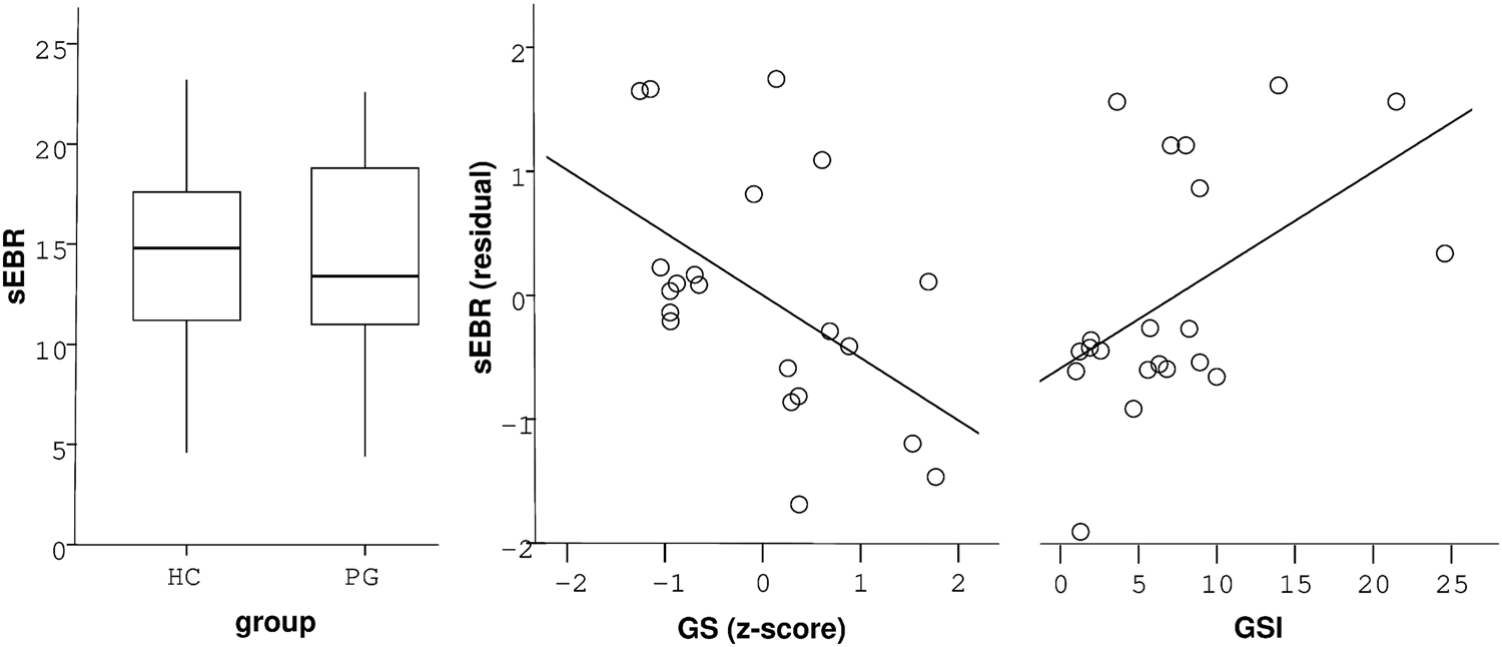
(**a**) SEBR did not differ in problem gamblers (PG) compared with healthy controls (HC). Vertical lines within boxplots represent the median, 25^th^, and 75^th^ percentile, respectively. Whiskers represent the range. (**b**) Multiple regression analysis revealed a negative correlation of gambling severity (GS, z-score) with sEBR in problem gamblers. (**c**) Overall psychopathology (GSI) was positively correlated with sEBR in gamblers.

SEBRs did not differ significantly between healthy controls (HC) and gamblers (G) (mean +/− SEM HC: 14.6 ± 1.1; G: 14.4 ± 1.4; F(1,39) = 0.02, p = 0.89, Fig. 1a). The Bayes factor quantifying the evidence for the null hypothesis similarly showed moderate evidence in favor of no group difference (BF_01_ = 3.24). Including only pathological gamblers (DSM-5 >  = 5), revealed a similar effect (PG: 13.4 ± 1.7; F(1,30) = 0.41, p = 0.53). In gamblers however, stepwise multiple regression analysis (adj. R² = 0.31, F(2,18) = 5.43, p = 0.01, Table 1, final model) revealed a negative correlation of sEBR with gambling severity (mean [95% CI]: β = −0.53 [−5.61 −0.69], p = 0.02, Fig. 1b) and a positive correlation with overall psychopathology (GSI) (β = 0.54 [0.1 0.78], p = 0.01, Fig. 1c). Age showed no significant effect on sEBR and was thus removed from the model.

**Table 1:** Regression results (gamblers only): Gambling severity (GS) and psychopathology (GSI) predicted sEBR in gamblers according to stepwise regression (final model).

	initial model	final model	alternative model
model statistics	adj. R² = 0.27 F(3,17) = 3.42, p = 0.04	adj. R² = 0.31, F(2,18) = 5.43, p = 0.01	adj. R² = 0.11 F(2,18) = 2.22, p = 0.14
GS	β = −0.54 [−5.88 −0.45] p = 0.03	β = −0.53 [−5.61 −0.69] p = 0.02	β = −0.47 [−5.68 0.09] p = 0.06
GSI	β = 0.55 [0.05 0.85] p = 0.03	β = 0.54 [0.1 0.78] p = 0.01	—
age	β = 0.008 [−0.35 0.36] p = 0.97	—	—
BDI-II	—	—	β = 0.31 [−0.08 0.34] p = 0.2

Age (initial model) was no significant predictor of sEBR. Depressive symptoms (BDI-II) instead of GSI scores did not explain significant variance in gamblers’ sEBR (alternative model). Values in brackets represent 95% confidence intervals.

Individual GSI scores were highly correlated with BDI-II scores (adj. R² = 0.78, F(1,19) = 71.31, p = 7.44 * 10^−8^). To exclude that the impact of psychopathology on gamblers’ sEBRs was exclusively driven by depressive symptoms, we computed another regression model that included BDI-II scores instead of participants GSI scores and gambling severity. This model did not explain a significant amount of variance of individual sEBRs (adj. R² = 0.09, F(2,18) = 2.0, p = 0.16, Table 1, alternative model) and BDI-II scores were not significantly associated with sEBRs (β = 0.31 [−0.09 0.38], p = 0.2).

**Figure 2:**
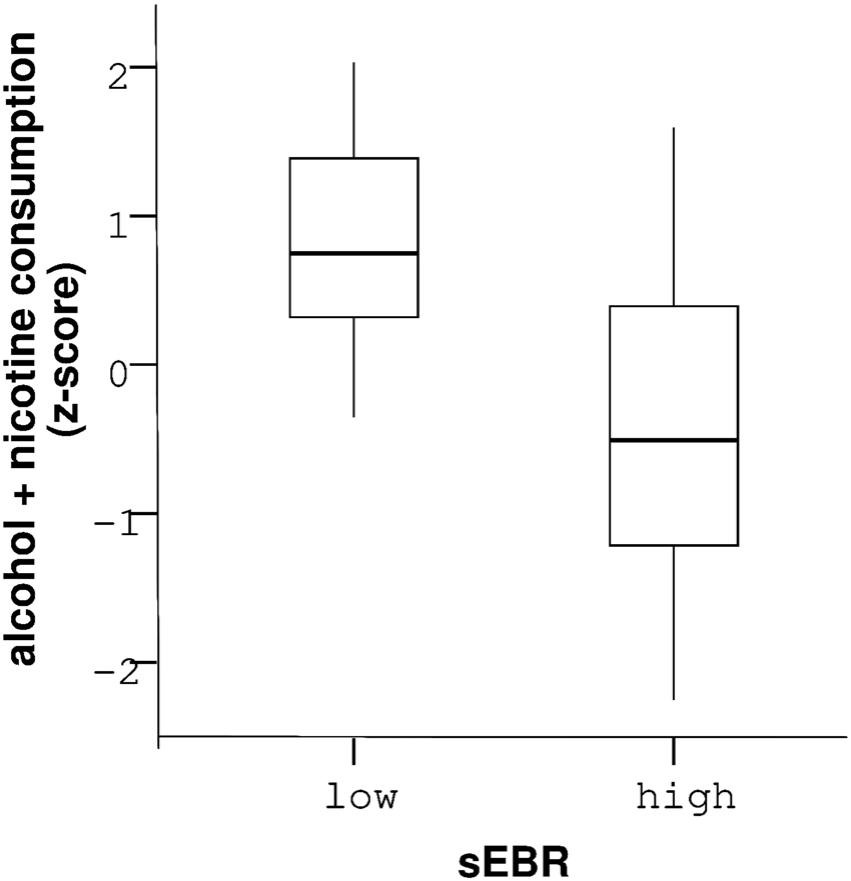
Healthy controls with low sEBR consumed more alcohol and nicotine (z-standardized) than control participants with high sEBR. Vertical lines within boxplots represent the median, 25^th^, and 75^th^ percentile, respectively. Whiskers represent the range of standardized consumption values.

In a further exploratory analysis (see *Statistical analyses*), we tested whether sEBR in healthy controls was associated with substance use (alcohol and nicotine consumption). For this purpose, we computed a compound score of alcohol and nicotine consumption and separated our control participants into a low sEBR and a high sEBR group according to a median split. Control participants with a low sEBR showed higher nicotine and alcohol consumption than participants with a high sEBR (F(1,18) = 5.92, p = 0.03, Fig. 2). In contrast to gamblers, GSI scores were not correlated with sEBRs in control participants (R² = 0.13, p = 0.11).

## Discussion

We tested for an association between problematic gambling behavior and spontaneous eye blink rate (sEBR) as a potential marker of striatal D2/3-receptor availability. We observed no differences in sEBR between problem gamblers and healthy controls. However, in gamblers, gambling severity was negatively associated with sEBR, suggesting a potential modulatory effect of striatal D2/3-receptor availability on the escalation of gambling behavior. Overall psychopathology, as assessed with the SCL-90-R questionnaire, was positively linked to sEBR in gamblers. An exploratory analysis revealed a negative association between sEBR and alcohol/nicotine use in healthy controls.

Our observation of similar sEBRs in gamblers and matched controls supports recent findings from direct assessments of striatal D2/3-receptor availability in pathological gamblers via PET^27,28^. Reduced striatal D2/3-receptor availability might therefore not constitute a risk factor for gambling disorder, in contrast to substance-based addictions, where reduced D2/3-receptor availability is a consistent finding in both animals^14,15^, and humans^8,49^. Likewise, reduced sEBR has been related to recreational cocaine use in humans^34^. Drug consumption presumably causes a higher subsequent increase in striatal dopamine levels than engagement in gambling. Thus, gambling may not suffice to compensate for reduced striatal D2/3-receptor availability as it is assumed for drug use. Noteworthy, recurrent abuse of stimulating substances can cause a decrease in striatal D2/3-receptor availability^23,50,51^. This aggravates the differentiation between cause and consequence regarding altered dopamine signaling in addiction disorders at least in cross-sectional studies in humans.

We found that in problem and pathological gamblers, gambling severity was negatively associated with sEBR. As a potential marker of striatal D2/3-receptor availability, this may indicate that gamblers with reduced D2/3-receptor availability are at higher risk for developing more severe gambling behavior. Interestingly, Clark *et al*.^27^ observed a negative association between D2/3-receptor binding potential in striatum and mood-related impulsivity in gamblers. Thus, lower striatal D2/3-receptor availability may be linked to the escalation of gambling behavior, possibly due to increased impulsivity in mood-intense states. This is further supported by a longitudinal study showing a clear link between an impulsive, negative-emotional personality and the development of gambling disorder^52^. Interestingly, Boileau *et al*.^23^ found a positive correlation between gambling severity and D3-receptor availability, as assessed via [^11^C]-(+)-PHNO binding potential, in substantia nigra (SN). They also revealed that [^11^C]-(+)-PHNO binding potential in SN negatively correlated with amphetamine induced dopamine release in striatum and that gamblers showed stronger amphetamine induced striatal dopamine release than controls^25^. This seems in line with recent evidence for a hyperdopaminergic state within striatum of pathological gamblers^24^. Taken together, our observed negative correlation of (presumably) striatal D2/3-receptor availability with gambling severity may at least partly translate into a positive association between striatal dopaminergic tone and severity of problematic gambling.

Overall psychopathology, as assessed via the GSI of the SCL-90-R screening, correlated positively with sEBR as a potential indicator of striatal dopamine transmission in gamblers, but not in healthy controls. Psychological aberrations such as psychoticism have previously been related to heightened sEBR^33,53^ and reduced striatal D2/3-receptor availability^46^. The consistent association of schizophrenia and increased striatal dopamine function^54–56^ together with a continuum model of psychosis^57^ further support the hypothesis that a psychosis-prone personality has a dopaminergic basis^58^. In healthy controls, the range of the GSI score was significantly lower than in problem and pathological gamblers with a maximum score of 1.1 compared to 2.46, respectively. This complements earlier observations of an association between mental health disorders and pathological gambling^59–61^, and may at least partly explain the absence of a correlation between GSI scores and sEBR in control participants. Note, however, that the GSI score is a coarse measure that incorporates a variety of different psychological aberrations, warranting caution in the interpretation of these findings.

In an exploratory analysis, we found that healthy controls displaying relatively low sEBR (i.e. potentially low striatal D2/3-receptor availability) consumed more alcohol and nicotine than participants with relatively high sEBR. This is in accordance with findings from PET studies showing reduced D2/3-receptor availability in alcohol and nicotine addiction^12,62–65^. Notably, alcohol and nicotine consumption both increase extracellular dopamine in striatum^63,66,67^ that likely leads to a downregulation of striatal dopamine signaling following chronic intake^12,64,65^. Thus, this finding might partly reflect a consequence of recurrent consumption, and should be further explored in larger samples.

Several limitations of the present study need to be acknowledged. First, the assumption of a positive correlation between sEBR and D2/3-receptor availability in striatum in humans is still debated. In a recent publication, Sescousse *et al*.^43^, report a negative relation between sEBR and dopamine synthesis capacity as assessed with [18 F]DOPA PET in a mixed sample of gamblers and control participants. Dang *et al*.^42^ found no significant correlation between D2/3-receptor availability and sEBR in humans. Notably, their sample of 20 subjects was quite heterogeneous in age (20–50 y) and body weight (<60–120 kg). As there is growing evidence that body weight is associated with D2/3-receptor availability^68^, this might have influenced their findings. Furthermore, PET imaging and sEBR assessment were separated on average by 17 months (3–32 months). Hence, more work is needed to clarify the exact relationship between sEBR and dopamine transmission in humans. Second, this was a cross-sectional study. Thus, we cannot exclude that higher sEBR in gamblers exhibiting more severe gambling may be a consequence of gambling history and corresponding adaptations in the dopaminergic system similar to observations in substance-based addiction. Third, only male participants were tested, limiting the generalization of the findings. Fourth, our sample of gamblers consisted predominantly of slot-machine and sports betting gamblers, thus limiting our conclusions to this particular subgroup of gamblers. Finally, the sample size was insufficient to examine potential differences between gambling subtypes that have for example been proposed in the “pathways model”^69^.

In light of the potential link between sEBR and striatal D2/3-receptor availability, our findings in gamblers and healthy controls indicate that attenuated striatal D2/3-receptor availability is not necessarily a risk factor for developing gambling disorder as postulated for substance-based addictions. Rather, attenuated striatal D2/3-receptor availability might aggravate engagement in gambling in problematic gamblers. One endophenotype of lower striatal D2/3-receptor availability is impulsivity^17^. Pathological gamblers suffering from relatively low striatal D2/3-receptor availability may be prone to an increased escalation of gambling behavior through attenuated cognitive control, heightened cue-reactivity and/or steeper delay discounting, specifically in emotionally-demanding states^15,59,70–72^.

Given that sEBR is an affordable and easily obtainable measure with a putative link to D2/3-receptor availability, it might be worthwhile to explore its applicability in clinical practice. For example, it would be of interest to explore sEBR as a potential predictor of treatment outcome in addiction and/or examine interindividual differences in sEBR changes post-treatment.

## Methods

### Subjects

21 male problem gamblers (#DSM-5 criteria >= 1), and 20 healthy male control subjects participated in this study. All gamblers reported regular gambling, and suffered from losing money while gambling. 12 gamblers fulfilled DSM-5 criteria (#criteria >= 4) of gambling disorder. Gamblers and healthy controls were matched for age, educational background, socioeconomic status, alcohol and nicotine consumption. Severity of gambling disorder was assessed by the ‘Kurzfragebogen zum Glücksspielverhalten’ (KFG)^73^, and a German adaptation of the South Oaks Gambling Screen (SOGS)^74^. Both questionnaires are validated screening tools for quantifying gambling disorder severity, and show good reliability (Cronbach’s Alpha: KFG: 0.79; SOGS: 0.97). Comprehensive demographic information is provided in Table 2. Participants were recruited via advertisements on local internet bulletin boards. Prior to enrollment in the study, phone interviews were conducted, and only gamblers who reported gambling on a regular basis, suffered from monetary loss, and fulfilled at least one of the DSM-5 criteria of pathological gambling were invited to participate. Gamblers were mainly engaged in slot machine gambling (67%), and sports betting (57%). A fraction also pursued (online) poker (14%), and roulette (14%). Eligible participants were interviewed by a psychologist to exclude a history of neurological or psychiatric disorders, current medication, and substance abuse other than nicotine and alcohol. All study procedures were approved by the local Institutional Review Board (Hamburg Board of Physicians). We confirm that all research was performed in accordance with relevant guidelines and regulations, and participants provided informed written consent prior to their participation. Participants received 10 EUR per hour as a compensation for participation.

**Table 2:** Sample description [mean ± standard deviation (min-max)]: Gamblers did not differ regarding age, income, years of education (YOE), alcohol (AUDIT) and nicotine consumption (^#^cigarettes), and eye blink rate (sEBR).

n	healthy controls	Gamblers	F/U	p
	20	21	—	—
age	26.4 ± 6.39 (19–45)	26.0 ± 6.66 (18–42)	F = 0.04	0.85
income	1028.15 ± 575.05 (0–2000)	1375.1 ± 819.51 (300–2700)	F = 2.44	0.13
YOE	11.75 ± 1.37 (9–14)	11.71 ± 1.82 (9–15)	F = 0.01	0.94
BDI-II	8.7 ± 8.3 (0–28)	15.1 ± 11.87 (2–42)	F = 3.96	0.05
GSI	0.32 ± 0.34 (0–1.1)	0.73 ± 0.62 (1–2.46)	F = 6.7	0.01
DSM-5	0.1 ± 0.38 (0–1)	5.1 ± 2.28 (1–8)	U = 1	1.1 * 10^−8^
KFG	1.45 ± 4.07 (0–18)	25.29 ± 14.54 (6–54)	U = 9	7.6 * 10^−8^
SOGS	0.4 ± 1.0 (0–4)	8.48 ± 4.61 (3–17)	U = 5.5	3.7 * 10^−8^
GS	−0.77 ± 0.21 (−0.85–0.08)	0.73 ± 0.86 (−0.38–2.3)	U = 217	8.2 * 10^−8^
AUDIT	6.75 ± 4.8 (0–15)	5.95 ± 6.98 (0–23)	F = 0.43	0.52
^#^cigarettes	9.25 ± 8.75 (0–30)	5.95 ± 6.76 (0–19)	U = 167.5	0.25
sEBR	14.6 ± 5.02 (4.6–23.2)	14.37 ± 5.08 (4.4–22.6)	F = 0.14	0.7

Gamblers displayed higher gambling severity (DSM-5, KFG, SOGS, sum of z-scores of KFG & SOGS (GS)), higher psychoticism (GSI), and a tendency for more depressive symptoms (BDI-II). Tests for group differences were based on ANOVA (F) for normally distributed variables, and Mann-Whitney U Tests otherwise.

### General procedure

Participants entered the lab in the afternoon around 2 pm. After they gave written informed consent, participants started with a five minutes sEBR assessment. Notably, sEBRs are stable throughout the day and rise in the evening^53^. Subsequently, they completed our lab’s standard questionnaire battery on the computer. On a separate testing day, participants performed two reward-based learning task in an fMRI setting. These data will be reported elsewhere.

### Psychological assessment

Following the sEBR assessment, participants completed questionnaires assessing gambling disorder severity (DSM-5 criteria, KFG, SOGS). In addition, participants completed the Symptom Check-List-90-R (SCL-90-R)^48^ that constitutes a screening tool for capturing current psychological pathology. As depressive symptoms are a common co-morbidity in pathological gambling^3^ participants also completed the Beck Depression Inventory (BDI-II)^75^. Within our standard questionnaire battery, subjects were also screened for any past or current psychiatric or neurological disease. We quantified nicotine consumption as self-reported number of cigarettes smoked per day. Alcohol use was measured via the Alcohol Use Disorders Identification Test (AUDIT)^76^.

### SEBR assessment

Spontaneous eye blink rates (SEBRs) were assessed via electromyography (EMG), utilizing a MP100 system running under the software Acqknowledge 3.9.1 (Biopac Systems, Goleta, California). Data was recorded via three Ag-AgCL electrodes with a sample rate of 1000 Hz, and an online bandpass filter of 28–500 Hz. One reference electrode was placed on the middle of the participant’s forehead and two electrodes were fixed below the lower lash line of the left eye, one of the electrodes centrically and the other one 2–3 mm in peripheral proximity. Participants sat in front of a computer screen and were instructed to move as little as possible while staring at a fixation cross at approximately 0.5 m distance for 5 minutes. A duration of 5 minutes has been shown to suffice for assessing stable mean sEBR values^77^. They were not explicitly told that sEBR was assessed and subjects were monitored during sEBR assessment to ensure that they were fixating the screen as instructed.

Individual sEBRs were computed using Matlab 2012b (MathWorks, Natick, MA) via the ‘*findpeaks’* function in a sliding window approach of 10 seconds to the acquired raw data. Blinks were defined as peaks exceeding the data’s mean within the moving window by six standard deviations. SEBRs were then calculated as average number of blinks per minute.

### Statistical analyses

All reported results were computed with PASW-SPSS-Statistics 17.0 (IBM Corporation, Somers, NY, USA). We utilized an ANOVA model to assess differences in sEBR between pathological gamblers and healthy controls. In addition, we calculated the Bayes factor in favor of the null hypothesis (BF_01_) via the JASP software package (Version 0.8.6, University of Amsterdam). Stepwise (backward elimination) multiple regression analysis was used to test the association between sEBR and gambling severity in gamblers. Gambling severity (GS) was computed as the mean of the two z-standardized gambling questionnaire scores (SOGS + KFG). Results were similar if only one score was used in the regression model. To control for overall psychopathology, the global severity index (GSI) of the SCL-90-R served as an additional predictor. We further controlled for age-related effects. In a second regression model, we controlled for individual depressive symptoms via individual BDI-II scores instead of GSI scores. We did not include both predictors in a single model due to their high correlation (R² = 0.78, p = 7.44 * 10^−8^).

In addition, we ran an exploratory analysis to test an association between sEBR and substance use in healthy controls based on the consistent finding in animals and humans, that low D2/3-receptor availability is a risk factor for developing SUD^12,13,78^. An individual substance use score was calculated as the sum of the z-standardized AUDIT questionnaire score and the z-standardized number of cigarettes smoked per day. Controls were separated into a low and a high sEBR group via a median split to test if the low sEBR group consumed more alcohol and /or nicotine according to the substance use score than the high sEBR group. Additionally, we computed a correlation analysis between sEBR and GSI scores in healthy controls.

Gaussianity, heteroscedasticity and absence of multicollinearity were tested for the respective analyses.

## Data Availability

All data will be made available upon request.
